# The Story of the Insane from Year to Year

**Published:** 1906-11-17

**Authors:** 


### THE STORY OF THE INSANE FROM YEAR TO YEAR.

Eighteenth Annual Series.

### EASTERN COUNTIES ASYLUM FOR IDIOTS AT COLCHESTER.

This institution contained 256 patients on the first of January, and at the end of the year the number had risen to 281. There were 46 admissions, and the total number under care during the year was 302. There were 5 discharges and 16 deaths. Of those admitted some were as young as three years and others as old as thirty-one. Of course many of them are most unpromising as regards chances of great improvement, but it is wonderful to notice how much has been done at this asylum to train boys and girls in some useful occupation; and at the present time not fewer than 139 boys and girls are receiving instruction in the workshops, and in some instances at least very creditable work is turned out. In the schoolrooms 69 have daily lessons. So much has this training been increased lately that Mr. Turner thinks that in the near future an extension of the comparatively new Packover schools will be required. We much regret to hear that there has been a falling off in the annual subscriptions; and that the Board finds that "as old subscribers pass away it becomes increasingly difficult to fill their places." In addition to this the report tells us that the outstanding liabilities on the Enlargement Fund amount to £1,200; and we further learn that the asylum is not provided with an isolation hospital for infectious disease. The latter drawback was thrown painfully to the front during the past year, when 85 cases of scarlet fever were under treatment. We venture very earnestly to urge on the inhabitants of East Anglia the desirability of giving donations to the Committee to build a small isolation hospital. We are aware that Lord Cadogan presided at a meeting held at Newmarket to obtain donations and subscriptions to the Enlargement Fund, and nearly £1,000 was thereby obtained, and we hope that this lead will be very generally followed throughout the counties most interested in the welfare of the asylum.

